# Mitochondrial translation defects and human disease

**DOI:** 10.20517/jtgg.2020.11

**Published:** 2020-05-23

**Authors:** Bryn D. Webb, George A. Diaz, Pankaj Prasun

**Affiliations:** Department of Genetics & Genomic Sciences, Icahn School of Medicine at Mount Sinai, New York, NY 10029, USA.

**Keywords:** Mitochondria, translation defect, tRNA, aminoacyl-tRNA synthetase, rRNA, ribosomal protein, mitochondrial disease, mtDNA

## Abstract

In eukaryotic cells, mitochondria perform the essential function of producing cellular energy in the form of ATP via the oxidative phosphorylation system. This system is composed of 5 multimeric protein complexes of which 13 protein subunits are encoded by the mitochondrial genome: Complex I (7 subunits), Complex III (1 subunit),Complex IV (3 subunits), and Complex (2 subunits). Effective mitochondrial translation is necessary to produce the protein subunits encoded by the mitochondrial genome (mtDNA). Defects in mitochondrial translation are known to cause a wide variety of clinical disease in humans with high-energy consuming organs generally most prominently affected. Here, we review several classes of disease resulting from defective mitochondrial translation including disorders with mitochondrial tRNA mutations, mitochondrial aminoacyl-tRNA synthetase disorders, mitochondrial rRNA mutations, and mitochondrial ribosomal protein disorders.

## INTRODUCTION

Mitochondria are double-membrane bound organelles found in most eukaryotic organisms with the important function of generating cellular energy via oxidative phosphorylation, but which also function in cellular signaling, cellular differentiation, cell death, and cell cycle regulation. Mitochondria are estimated to be comprised of approximately 1100 proteins and are unique organelles in that they have their own genome and ribosomes that carry out protein synthesis inside the mitochondria^[1]^. The mitochondrial genome, which is housed in the mitochondrial matrix, encodes 37 genes: 13 which encode protein subunits of respiratory Complexes I, III, IV, and V; 22 which encode mitochondrial tRNAs; and 2 which encode mitochondrial rRNAs. By far the majority of mitochondrial proteins are produced on cytosolic ribosomes and are transported to the mitochondria as precursors via the translocase of the mitochondrial outer membrane (TOM complex), the presequence translocase (TIM23 complex), and presequence-translocase-associated motor located at the inner mitochondrial membrane^[2]^.

Oxidative phosphorylation and generation of cellular ATP requires coordinated biogenesis and assembly of respiratory chain complexes at the inner mitochondrial membrane. Electrons are transferred along the respiratory chain complexes from the reducing equivalents NADH and FADH2 to oxygen to produce water and generate a proton gradient across the inner membrane. This proton gradient enables ATP synthase to generate ATP from ADP and phosphate. In humans, five multi-subunit protein complexes compose the respiratory chain and oxidative phosphorylation system: NADH dehydrogenase (Complex I); succinate dehydrogenase (Complex II); coenzyme Q: cytochrome c-oxidoreductase (Complex III); cytochrome c oxidase (Complex IV); and ATP synthase (Complex V). Complex II is composed of proteins encoded entirely by the nuclear genome, whereas the remaining complexes have protein subunit components encoded by both nuclear and mitochondrial genomes. Additionally, complex assembly is a highly coordinated process involving a number of assembly factors, as well as coordination of nuclear and mitochondrial genes. Defects in mitochondrial translation processes may result in impaired activities of these complexes, resulting in deficient aerobic energy metabolism and clinical disease in humans^[3]^.

Mitochondrial translation is specifically defined as the process within mitochondria whereby mitochondrial mRNA (mt-mRNA) is translated by mitochondrial ribosomes (mitoribosomes) to generate an amino acid polypeptide. Mitochondrial translation is necessary for the generation of thirteen respiratory complex subunits. mt-mRNAs are unique in that they are uncapped, have no or very few 5′-untranslated nucleotides, and contain a poly A tail that immediately follows or forms part of the stop codon^[4]^. The mitoribosome translates the mt-mRNA by inducing the binding of complementary tRNA anticodon sequences to mt-mRNA codons in a manner analogous to that performed by cytoplasmic ribosomes. The tRNAs carry specific amino acids that are linked together into a polypeptide as the mt-mRNA passes through and is read by the mitoribosome. Mitoribosomes have a higher protein:RNA ratio (2:1 *vs*. 1:2 in cytoplasmic ribosomes) and are less dense (55S *vs*. 80S) than cytoplasmic ribosomes^[5]^. Additionally, mitoribosomal translation is unique in that there are several differences from the universal genetic code. Human mitochondria translate the conventional UGA stop codon as tryptophan, reprogram the two conventional arginine codons AGA and AGG for termination, and code the conventional isoleucine AUA codon as methionine^[6]^.

Structural studies have established that many mitochondrial ribosome proteins have eubacterial orthologs, but there also exist additional proteins without such orthologs. Mitoriboproteins have traditionally been named by a MRPS (Mitochondrial Ribosomal Protein Small subunit)/MRPL (Mitochondrial Ribosomal Protein Large subunit) nomenclature^[5]^. Recently, a new naming convention has been proposed based on functional/structural relationships of mitoribosomal proteins across species in order to reduce ambiguity arising from non-orthologous proteins from different species being assigned similar names^[7]^.

Mitochondrial translation defects resulting in human disease may have varying organ involvement, varying age of onset, and varying modes of inheritance. This specific class of mitochondrial disease may be caused by the following mechanisms: mitochondrial tRNA mutations, mitochondrial aminoacyl-tRNA synthetase mutations, mitochondrial rRNA mutations, and mitochondrial ribosomal protein mutations. Additional mechanisms of abnormal mitochondrial translation exist, including impaired translation secondary to mtDNA depletion and defects in mitochondrial RNA synthesis, modification, and degradation, which are beyond the scope of this article but have been recently reviewed^[8,9]^.

## MITOCHONDRIAL tRNA MUTATIONS

All 22 mt-tRNAs are encoded by the mitochondrial genome, and the primary function of mt-tRNAs is to deliver amino acids to the nascent polypeptide chain during mitochondrial protein translation. Mitochondrial tRNAs are truncated when compared to their canonical cytosolic tRNA counterparts, and, in some cases, such as in tRNA^Ser(AGY)^, one arm of the classic cloverleaf secondary structure of tRNA is lost^[10]^.

The first report of a mt-tRNA mutation causing human disease was published in 1990 when Kobayashi *et al.*^[11]^ revealed that a mutation in the mitochondrial tRNA^Leu^ gene (*MTTL1*) was causative of mitochondrial myopathy, encephalopathy, lactic acidosis, and stroke-like episodes (MELAS)^[11,12]^. Since then, over 300 mutations in mt-tRNA genes have been identified to cause human disease [Table 1]. Most of these mutations prevent tRNA aminoacylation. mt-tRNA mutations have been identified in various structural locations including in the anti-codon wobble position, anti-codon stem, acceptor stem, DHU stem, TYC stem, and the variable loop^[12]^. Disorders associated with mitochondrial t-RNA mutations are summarized in Table 1.

Interestingly, different point mutations in the same mt-tRNA molecule can result in different human diseases. For example, the point mutation m.14709T>C in *MTTE* (gene that encodes the mitochondrial tRNA^Glu^) can result in the phenotype of maternally inherited diabetes and deafness, whereas the point mutations m.14674T>G or m.14674T>C in *MTTE* can result in infantile transient mitochondrial myopathy.

Nearly all mitochondrial disease resulting from mt-tRNA mutations display maternal inheritance as mitochondrial DNA is inherited from the mother. However, few instances of paternal inheritance have been reported as well^[13,14]^. Cells that carry a homogeneous population of the mitochondrial genome, either wild-type or mutant, are termed homoplasmic. Cells that carry two or more populations of the mitochondrial genome are termed heteroplasmic. With mitochondrial disease due to maternal inheritance, clinical disease severity often correlates with mutation load in affected tissues.

The two most well-known mitochondrial diseases associated with mt-tRNA mutations are MELAS and myoclonic epilepsy with ragged red fibers (MERRF). In approximately 80% of MELAS patients, the causative mutation is the m.3243A>G pathogenic variant in *MTTL*_*1*_ (mt-tRNA^Leu^). Most patients with MELAS develop symptoms between ages 2 and 40 years old, and these symptoms include stroke-like episodes, encephalopathy with seizures and/or dementia, muscle weakness, exercise intolerance, headaches, vomiting, hearing impairment, peripheral neuropathy, learning disability, and short stature. Treatment for MELAS is supportive and includes treatment with a mitochondrial cocktail. Intravenous arginine is recommended during acute stroke-like episodes, and arginine should be given orally for prophylaxis after a patient has had a first stroke-like episode^[15]^.

The most common mutation causing MERRF in more than 80% of affected patients is the m.8344A>G mutation in *MTTK* (mt-tRNA^Lys^). Onset of MERRF is usually in childhood and the first symptom is often myoclonus. Other common symptoms and findings are epilepsy, ataxia, weakness, dementia, hearing loss, short stature, optic atrophy, and cardiomyopathy with Wolff-Parkinson-White syndrome. Treatment is also supportive with antiepileptic medications to treat seizures, mitochondrial cocktail, and physical therapy^[16]^.

## MITOCHONDRIAL AMINOACYL-tRNA SYNTHETASE DISORDERS

Mitochondrial aminoacyl-tRNA synthetases (mt-ARSs) are essential for protein synthesis in the mitochondria and generation of oxidative phosphorylation (OXPHOS) system components. mt-ARS proteins are nuclear-encoded and function to charge mitochondrial tRNA molecules, which are mitochondrial-encoded, with their cognate amino acids. While mt-ARS proteins vary in size and oligomeric state (from monomer to tetramer), all contain a catalytic domain and a tRNA anticodon-binding domain^[17]^. mt-ARS genes are named with an *ARS2* nomenclature (for example, *MARS2* for methionine tRNA synthetase, mitochondrial). For the amino acids glycine and lysine, a separate mt-ARS gene does not exist, and *GARS* and *KARS*, respectively, function as the aminoacyl-tRNA synthetase in both the cytosol and the mitochondria. Additionally, an mt-ARS has not been identified for glutamine (Q), and Q-tRNA is believed to be formed by postconjugation modification of glutamate^[18]^.

The first Mendelian disease reported to be caused by mt-ARS mutations was leukoencephalopathy with brain stem and spinal cord involvement and lactate elevation (MIM #611105) due to autosomal recessive pathogenic variants in *DARS2*, which was reported in 2007^[19]^. Since then, pathogenic variants in all known mt-ARSs have been identified with the majority being identified by whole exome sequencing studies, and the associated conditions represent a new class of Mendelian disorders [Table 2]. All mt-ARS disorders exhibit autosomal recessive inheritance and most often patients are compound heterozygotes^[17]^. These Mendelian disorders are extremely rare as deleterious mutations in mt-ARS genes leading to absent mt-ARS function are expected to be lethal. Therefore, patients most often have at least one allele with a mild mutation leading to some residual mt-ARS gene function.

Interestingly, although pathogenic variants in all mt-ARSs are expected to result in disruption of protein synthesis of OXPHOS system components via impairment of mitochondrial translation, the identified mt-ARS disorders each display strikingly specific clinical phenotypes with specific tissue involvement [Table 2]^[17,20,21]^. Most frequently, mt-ARS disorders display central nervous system involvement, but additional organ systems are specifically involved in certain disorders, such as ovaries in the case of *HARS2* and *LARS2* or kidney in the case of *SARS2* [Table 2]^[22–24]^. Additionally, age of onset is highly variable for the various mt-ARS disorders. The molecular mechanisms behind this selective tissue involvement and disease phenotype for specific mt-ARS disorders are currently poorly understood.

A wide variety of neurological symptoms is also seen with mt-ARS disorders. Leukoencephalopathy may be seen with *AARS2*, *DARS2*, *EARS2*, *NARS2*, *PARS2*, and *WARS2* disorders. Epilepsy may be seen with *CARS2*, *EARS2*, *FARS2*, *NARS2*, *PARS2*, *RARS2*, *TARS2*, *VARS2*, and *WARS2* disorders. Peripheral neuropathy is seen with *IARS2* disorder. Sensorineural hearing loss may be seen with *HARS2*, *IARS2*, *LARS2*, *MARS2*, *NARS2*, and *PARS2* disorders^[18]^.

Pathogenic mutations in *GARS*, which functions in both the cytosol and mitochondria, may cause autosomal dominant Charcot-Marie-Tooth disease, type 2D (MIM #601472) or autosomal dominant neuropathy, distal hereditary motor, type VA (MIM #600794)^[25]^. Additionally, a few cases of *GARS* variants causing autosomal recessive disease have been reported leading to cardiomyopathy or complex neurological phenotypes [Table 2]. Pathogenic mutations in *KARS* may cause autosomal recessive Charcot-Marie-Tooth disease, recessive intermediate B (MIM #613641) or deafness, autosomal recessive 89 (MIM #613916)^[26,27]^. Interestingly, Ruzzenente *et al*.^[28]^ recently reported a patient with compound heterozygous *KARS* variants leading to impaired mitochondrial translation, but intact cytosolic translation. This patient had symptoms of sensorineural deafness, developmental delay, hypotonia, and lactic acidosis^[28]^. Additional case reports have described additional various phenotypes for patients with pathogenic *KARS* mutations including optic neuropathy, progressive leukoencephalopathy, and cardiomyopathy, among others^[29–31]^.

Failure of charging of glutaminyl mt-tRNA (mt-tRNA^Gln^) has also been identified to cause disease. The GatCAB aminoacyl-tRNA amidotransferase complex provides this function and is composed of three subunits: GATA encoded by *QRSL1*, GATB encoded by *GATB*, and GATC encoded by *GATC*. Patients with defects in glutaminyl mt-tRNA charging present in infancy with lethal cardiomyopathy and lactic acidosis. Pathogenic variants have been identified in *QRSL1*, *GATB*, and *GATC*, and all cause autosomal recessive disease^[32,33]^.

In addition to mt-ARS genes functioning in mitochondrial translation, there is growing evidence that mt-ARS proteins have potential non-canonical roles in immune regulation, inflammation, and neuronal differentiation^[34]^. Further work is in progress to further explore the many roles of mt-ARS genes.

## MITOCHONDRIAL rRNA MUTATIONS

Mitochondrial 55S ribosomes are composed of two subunits. The small 28S subunit (mtSSU) functions to catalyze the peptidyl-transferase reaction and the large 39S subunit (mtLSU) functions in mt-mRNA binding and decoding^[8]^. The 28S and 39S mitochondrial ribosome subunits are composed of 12S mt-rRNA (mtSSU) and 16S mt-rRNA (mtLSU) and ribosomal proteins. Both mt-rRNAs are processed from the polycistronic heavy strand transcript, which also encodes tRNA^Phe^ and tRNA^Val^. Following release of the mature mt-rRNAs by endonucleolytic cleavage, assembly of the functional mitoribosome proceeds via a complex process involving maturation and processing of mt-rRNAs and association with ribosomal proteins^[35]^. In addition to the 16S mt-rRNA, the large subunit of mammalian ribosomes also include tRNA^Phe^ or tRNA^Val[36,37]^.

The gene *MTRNR1* encodes the mitochondrial 12S ribosomal RNA, and the gene *MTRNR2* encodes the mitochondrial 16S ribosomal RNA. Mutations in *MTRNR1* are associated with hearing impairment with or without aminoglycoside exposure. The *MTRNR1* mutations m.1555A>G^[38]^ and m.1494C>T^[39]^ have been described as a cause of maternally inherited deafness in numerous case reports but the phenotype is variable and not completely penetrant. The identification of a pedigree in which deafness manifested when the m.1555A>G variant was co-inherited with a loss-of-function *SSBP1* variant suggests that SSBP1 may be a phenotypic modifier of m.1555A>G-associated deafness^[40]^. Additional examples of complex phenotypes involving m.1555A>G include in a pedigree in which the hearing loss co-segregated with familial dilated cardiomyopathy due to mutations in *MT-ATP6*^[41]^. Recently, expansion of the *MTRNR1* poly-cytidine tract at m.961 has been reported to be associated with non-ophthalmologic manifestations (intellectual disability, epilepsy, and migraine) in a kindred also segregating Leber’s hereditary optic neuropathy due to m.3460G>A, although this association is not statistically validated^[42]^. In contrast to the numerous reports of human disease-associated variants in *MTRNR1*, only a single variant in *MTRNR2*, m.2336C>T, has been identified as a cause of hypertrophic cardiomyopathy in humans^[43]^.

## MITOCHONDRIAL RIBOSOMAL PROTEIN DISORDERS

Mammalian mitoribosomes are composed of rRNA and mitochondrial ribosomal protein components. In humans, 30 mitochondrial ribosomal small subunit proteins (MRPSs) assemble with the 12S mt-rRNA to form the small mitoribosomal 28S subunit. Similarly, 50 mitochondrial ribosomal large subunit proteins (MRPLs) assemble with the 16S mt-rRNA along with tRNA to form the large mitoribosomal 39S subunit^[44]^. MRPS and MRPL proteins are all encoded by the nuclear genome.

At present, nine *MRPS* genes and three *MRPL* genes have been identified to cause mitochondrial disease in humans [Table 3]. All are inherited in an autosomal recessive fashion. Mutations in mitochondrial ribosomal protein genes may destabilize the mitoribosomal subunits impacting translation, as has been shown via proteomic analysis with *MRPS34* disorder^[44]^. Despite the presumptive shared pathogenesis of destabilizing either the large or small mitoribosomal subunits, the clinical phenotypes associated with mutations in genes encoding mitoribosomal structural proteins are surprisingly diverse [Table 3]. Most of these disorders present early in life, although missense mutations in *MRPS22* can present with ovarian failure in adolescent females^[45]^. Neurological deficits have been observed in the majority of patients with this subset of disorders but additional associated clinical phenotypes include hepatopathy, renal dysfunction, deafness, myopathy, and craniofacial or cardiac phenotypes. The neurological features may be variable and range from structural lesions such as agenesis of the corpus callosum to classical Leigh syndrome or functional deficits without apparent structural lesions. Most mitochondrial ribosomal protein subunit disorders cause severe disease often with multi-organ involvement and early death. In the future, additional mitochondrial ribosomal protein disorders are highly likely to be identified via whole exome sequencing of patients with suspected mitochondrial disease.

## CONCLUSION

Defects in mitochondrial translation may result in a vast array of clinical disease. Disease mechanisms include, but are not limited to, mitochondrial tRNA mutations, mitochondrial aminoacyl-tRNA synthetase mutations, mitochondrial rRNA mutations, and mitochondrial ribosomal protein mutations. Understanding disease biology of these mitochondrial translation defects is a necessary predecessor to developing effective treatment for these disorders. More research is necessary to further understand this emerging class of mitochondrial disease.

## Figures and Tables

**Table 1.** Clinical phenotypes of mt-tRNA disorders

Gene	Alternative name	Clinical phenotype(s)	PMID**
*MTTA*	mt-tRNA-Ala	Myotonic dystrophy-like myopathy; mitochondrial myopathy	14569122; 16476954
*MTTC*	mt-tRNA-Cys	MELAS; dystonia	8829635; 9185178; 17724295
*MTTD*	mt-tRNA-Asp	Myopathy	16059939
*MTTE*	mt-tRNA-Glu	MIDD; transient infantile mitochondrial myopathy	15048886; 19720722
*MTTF*	mt-tRNA-Phe	MELAS; MERRF; myopathy; epilepsy; encephalopathy; tubulointerstitial nephropathy	9771776; 15184630; 16769874; 11231339
*MTTG*	mt-tRNA-Gly	Hypertrophic cardiomyopathy; exercise intolerance; sudden death	8079988; 11971101; 8888049
*MTTH*	mt-tRNA-His	Cardiomyopathy; RP; MERRF; MELAS; NSHL	11038324; 12682337; 14967777; 21931169
*MTTI*	mt-tRNA-lle	Cardiomyopathy; familial hypertrophic cardiomyopathy; CPEO	1978914; 11782991; 20149659
*MTTK*	mt-tRNA-Lys	MERRF; cardiomyopathy and deafness; neurogastrointestinal encephalomyopathy; MIDD; progressive external ophthalmoplegia with myoclonus	2112427; 2124116; 8651277; 9380435; 9571188; 10220860
*MTTL1*	mt-tRNA-Leu (UUR)	MELAS; MERRF; cardiomyopathy with or without skeletal myopathy; encephalopmyopathy; CPEO; Kearns-Sayre syndrome; sudden infant death syndrome; Leigh syndrome; MIDD; SNHL; FSGS	2102678; 2268345; 8254046; 7906985; 8111377; 8265770; 10519336; 11448301
*MTTL2*	mt-tRNA-Leu (CUN)	Encephalomyopathy; myopathy; cardiomyopathy	8923013; 9012410; 11313776
*MTTM*	mt-tRNA-Met	Myopathy	9633749
*MTTN*	mt-tRNA-Asn	CPEO; myopathy	8254046; 7980504
*MTTP*	mt-tRNA-Pro	Myopathy; MERFF	7689388; 19273760
*MTTQ*	mt-tRNA-Gln	Myopathy; sensorineural deafness and migraine; MELAS	10996779; 11424923; 11171912
*MTTR*	mt-tRNA-Arg	Encephalomyopathy	15286228; 19809478
*MTTS1*	m-tRNA-Ser (UCN)	MERRF; MELAS; palmoplantar keratoderma with deafness; NSHL; exercise intolerance	7669057; 8019558; 10978361; 14605505
*MTTS2*	mt-tRNA-Ser (AGY)	Cerebellar ataxia, cataract, and diabetes mellitus; MERRF; MELAS	9792552; 16950817
*MTTT*	mt-tRNA-Thr	Fatal infantile myopathy; myopathy	1645537; 28187756; 30236074
*MTTV*	mt-tRNA-Val	Ataxia, progressive seizures, mental deterioration, and hearing loss; Leigh syndrome; hypertrophic cardiomyopathy; MELAS	9443499 ; 9450773; 11799391; 15465092; 21986556
*MTTW*	mt-tRNA-Trp	Encephalopathy; myopathy; neurogastrointestinal syndrome; encephalocardiomyopathy; Leigh syndrome	7695240; 9673981; 15054399; 18337306; 12776230
*MTTY*	mt-tRNA-Tyr	Exercise intolerance; CPEO with myopathy; FSGS and dilated cardiomyopathy	11071502; 11756614; 14598342

MELAS: mitochondrial encephalomyopathy, lactic acidosis, and stroke-like episodes; MIDD: maternally inherited diabetes and deafness; MERRF: myoclonic epilepsy with ragged red fibers; RP: retinitis pigmentosa; NSHL: nonsyndromic hearing loss; CPEO: chronic progressive external ophthalmoplegia; SNHL: sensorineural hearing loss; FSGS: focal segmental glomerulosclerosis.

* Seminal works highlighted including first reports of a gene causing human disease as well as key reports of new phenotypes. References: OMIM (https://omim.org) and MitoMap (https://www.mitomap.org)

**Table 2.** Clinical phenotypes of mt-ARS disorders

Gene	Mutation type	Inheritance	OMIM phenotype	Main organ(s) affected	OMIM Phenotype	Age at onset	PMID^#^
*AARS2*	SNV	Recessive	614096	Heart	Hypertrophic cardiomyopathy	Infancy	21549344
*AARS2*	SNV	Recessive	615889	Brain, ovaries	Progressive leukoencephalopathy with ovarian failure	Childhood-adulthood	2480803
*CARS2*	SNV	Recessive	616672	Brain, muscle	Combined oxidative phosphorylation deficiency, 27	Neonatal-childhood	25361775
*DARS2*	SNV	Recessive	611105	Brain	Leukoencephalopathy with brainstem and spinal cord involvement and lactate elevation	Childhood-adulthood	17384640
*EARS2*	SNV	Recessive	614924	Brain	Combined oxidative phosphorylation deficiency, 12	Infancy	22492562
*FARS2*	SNV	Recessive	614946	Brain	Combined oxidative phosphorylation deficiency, 14	Infancy	22499341
*FARS2*	SNV	Recessive	617046	Bran	Spastic paraplegia 77, autosomal recessive	Infancy-childhood	26553276
*HARS2*	SNV	Recessive	614926	Cochlea, ovaries	Perrault syndrome, 2	Childhood-adulthood	21464306
*IARS2*	SNV	Recessive	616007	Brain, bone, eyes	Cataracts, growth hormone deficiency, sensory neuropathy, sensiorineural hearing loss, and skeletal dysplasia	Infancy	25130867
*LARS2*	SNV	Recessive	615300	Cochlea, ovaries	Perrault syndrome, 4	Childhood-adulthood	23541342
*LARS2*	SNV	Recessive	617021	Brain, blood	Hydrops, lactic acidosis, and sideroblastic anemia	Neonatal	26537577
*MARS2*	CNV	Recessive	611390	Brain	Spastic ataxia 3, autosomal recessive	Childhood-adulthood	22448145
*MARS2*	SNV	Recessive	616430	Brain, muscle	Combined oxidative phosphorylation deficiency, 25	Infancy	25754315
*NARS2*	SNV	Recessive	616239	Brain, muscle, cochlea	Combined oxidative phosphorylation deficiency, 24	Infancy	25385316
*NARS2*	SNV	Recessive	618434	Cochlea	Deafness, autosomal recessive 94	Infancy	25807530
*PARS2*	SNV	Recessive	618437	Brain	Epilepetic encephalopathy, early infantile, 75	Neonatal-infancy	25629079
*RARS2*	SNV	Recessive	611523	Brain	Pontocerebellar hypoplasia, type 6	Infancy-childhood	17847012
*SARS2*	SNV	Recessive	613845	Kidney	Tubulopathy (hyperuricemia, metabolic alkalosis), pulmonary hypertension, and progressive renal failure (HUPRA syndrome)	Infancy	21255763
*TARS2*	SNV	Recessive	615918	Brain, muscle	Combined oxidative phosphorylation deficiency, 21	Neonatal	24827421
*VARS2*	SNV	Recessive	615917	Brain, muscle	Combined oxidative phosphorylation deficiency, 20	Infancy	25058219
*WARS2*	SNV	Recessive	617710	Brain, muscle	Neurodevelopmental disorder, mitochondria, with abnormal movements and lactic acidosis, with or without seizures	Infancy	28236339
*YARS2*	SNV	Recessive	613561	Muscle, blood	MLASA	Infancy-childhood	20598274
GARS*	SNV	Dominant	601472	Nerves	Charcot Marie Tooth disease, type 2D	Adulthood	12690580
GARS*	SNV	Dominant	600794	Nerves	Neuronopathy, distal hereditary motor, type VA	Adulthood	12690580
GARS*	SNV	Recessive	N/A	Brain, heart	Cardiomyopathy or growth retardation and complex neurological presentation	Neonatal-childhood	25058219; 24669931; 28675565
KARS*	SNV	Recessive	613641	Nerves	Charcot Marie Tooth disease, recessive intermediate, B	Childhood	20920668
KARS*	SNV	Recessive	613916	Cochlea	Deafness, autosomal recessive 89	Infancy-childhood	23768514

mt-ARS: mitochondrial aminoacyl-tRNA synthetases; MLASA: myopathy, lactic acidosis, and sideroblastic anemia; SNV: single nucleotide variation; CNV: copy number variation; HUPRA: Hyperuricemia, pulmonary hypertension, renal failure, and alkalosis.

* *GARS* and *KARS* function in both the cytosol and mitochondria;

# seminal works highlighted including first reports of a gene causing human disease as well as key reports of new phenotypes. References: OMIM (https://omim.org)

**Table 3.** Clinical phenotypes of mitochondrial ribosomal protein disorders

Gene	Inheritance	OMIM number	OMIM phenotype	Clinical phenotype	Age at onset	PMID*
Small subunit						
*MRPS2*	Recessive	617950	Combined oxidative phosphorylation deficiency, 36	Developmental delay, hypoglycemia, lactic acidemia, sensorineural hearing loss	Infancy	29576219
*MRPS7*	Recessive	617872	Combined oxidative phosphorylation deficiency, 34	Lactic acidemia, hepatorenal failure, sensorineural deafness	Infancy	25556185
*MRPS14*	Recessive	618378	Combined oxidative phosphorylation deficiency, 38	Hypertrophic cardiomyopathy, growth retardation, hypotonia, lactic acidemia, dysmorphism, intellectual disability	Newborn	30358850
*MRPS16*	Recessive	610498	Combined oxidative phosphorylation deficiency, 2	Agenesis of corpus callosum, brachydactyly, dysmorphism, lactic acidemia	Newborn	15505824
*MRPS22*	Recessive	611719	Combined oxidative phosphorylation deficiency, 5	Dysmorphism, hypotonia, hyperammonemia, lactic acidemia, renal tubulopathy, hypertrophic cardiomyopathy, cardiac septal defects	Newborn	17873122; 21189481; 25663021; 28752220
*MRPS22*	Recessive	618117	Ovarian dysgenesis, 7	Ovarian dysgenesis	Adolescence	29566152; 31042289
*MRPS23*	Recessive	N/A	N/A	Hepatic disease, combined oxidative phosphorylation deficiency	Childhood	26741492
*MRPS28*	Recessive	N/A	N/A	Craniofacial dysmorphism, developmental delay, intrauterine growth retardation	Infancy	30566640
*MRPS34*	Recessive	617664	Combined oxidative phosphorylation deficiency, 32	Dysmorphism, hypotonia, hyperammonemia, lactic acidemia, renal tubulopathy, hypertrophic cardiomyopathy, cardiac septal defects	Infancy	28777931
*MRPS39 (PTCD3)*	Recessive	N/A	N/A	Intrauterine growth retardation, Leigh syndrome, optic atrophy	Infancy	30607703
Large Subunit						
*MRPL3*	Recessive	614582	Combined oxidative phosphorylation deficiency, 9	Hypoglycemia, hypertrophic cardiomyopathy, intellectual disability, lactic acidemia, liver fibrosis, renal tubulopathy, sensorineural hearing loss	Infancy	27815843; 21786366
*MRPL12*	Recessive	N/A	N/A	Dysmorphism, hypotonia, intrauterine and postnatal growth retardation, intellectual disability, lactic acidemia, nystagmus, cerebellar ataxia, basal ganglia/white matter MRI hyperintensities	Infancy	23603806
*MRPL44*	Recessive	615395	Combined oxidative phosphorylation deficiency, 16	Hypertrophic cardiomyopathy, lactic acidemia, liver steatosis	Infancy	23315540

* Seminal works highlighted including first reports of a gene causing human disease as well as key reports of new phenotypes. References: OMIM (https://omim.org)

## References

[1] Pagliarini DJ, Calvo SE, Chang B, Sheth SA, Vafai SB, A mitochondrial protein compendium elucidates complex I disease biology. Cell 2008;134:112–23. 18614015 10.1016/j.cell.2008.06.016 PMC2778844

[2] Priesnitz C, Becker T. Pathways to balance mitochondrial translation and protein import. Genes Dev 2018;32:1285–96. 30275044 10.1101/gad.316547.118 PMC6169841

[3] Battersby BJ, Richter U. Why translation counts for mitochondria -retrograde signalling links mitochondrial protein synthesis to mitochondrial biogenesis and cell proliferation. J Cell Sci 2013;126:4331–8. 24013545 10.1242/jcs.131888

[4] Smits P, Smeitink J, van den Heuvel L. Mitochondrial translation and beyond: processes implicated in combined oxidative phosphorylation deficiencies. J Biomed Biotechnol 2010;2010:737385. 20396601 10.1155/2010/737385 PMC2854570

[5] Lightowlers RN, Rozanska A, Chrzanowska-Lightowlers ZM. Mitochondrial protein synthesis: figuring the fundamentals, complexities and complications, of mammalian mitochondrial translation. FEBS Lett 2014;588:2496–503. 24911204 10.1016/j.febslet.2014.05.054 PMC4099522

[6] Richter R, Pajak A, Dennerlein S, Rozanska A, Lightowlers RN, Translation termination in human mitochondrial ribosomes. Biochem Soc Trans 2010;38:1523–6. 21118119 10.1042/BST0381523

[7] Ban N, Beckmann R, Cate JH, Dinman JD, Dragon F, A new system for naming ribosomal proteins. Curr Opin Struct Biol 2014;24:165–9. 24524803 10.1016/j.sbi.2014.01.002 PMC4358319

[8] Van Haute L, Pearce SF, Powell CA, D’Souza AR, Nicholls TJ, Mitochondrial transcript maturation and its disorders. J Inherit Metab Dis 2015;38:655–80. 26016801 10.1007/s10545-015-9859-z PMC4493943

[9] Barchiesi A, Vascotto C. Transcription, processing, and decay of mitochondrial RNA in health and disease. Int J Mol Sci 2019;20. 10.3390/ijms20092221 PMC6540609 31064115

[10] Gonzalez-Serrano LE, Chihade JW, Sissler M. When a common biological role does not imply common disease outcomes: disparate pathology linked to human mitochondrial aminoacyl-tRNA synthetases. J Biol Chem 2019;294:5309–20. 30647134 10.1074/jbc.REV118.002953 PMC6462531

[11] Kobayashi Y, Momoi MY, Tominaga K, Momoi T, Nihei K, A point mutation in the mitochondrial tRNA(Leu)(UUR) gene in MELAS (mitochondrial myopathy, encephalopathy, lactic acidosis and stroke-like episodes). Biochem Biophys Res Commun 1990;173:816–22. 2268345 10.1016/s0006-291x(05)80860-5

[12] Abbott JA, Francklyn CS, Robey-Bond SM. Transfer RNA and human disease. Front Genet 2014;5:158. 24917879 10.3389/fgene.2014.00158 PMC4042891

[13] Luo S, Valencia CA, Zhang J, Lee NC, Slone J, Biparental inheritance of mitochondrial DNA in humans. Proc Natl Acad Sci U S A 2018;115:13039–44. 30478036 10.1073/pnas.1810946115 PMC6304937

[14] Schwartz M, Vissing J. Paternal inheritance of mitochondrial DNA. N Engl J Med 2002;347:576–80. 12192017 10.1056/NEJMoa020350

[15] El-Hattab AW, Almannai M, Melas SF. In: Adam MP, Ardinger HH, Pagon RA, Wallace SE, editors. GeneReviews (R). Seattle (WA); 1993.

[16] DiMauro S, Hirano M. Merrf. In: Adam MP, Ardinger HH, Pagon RA, Wallace SE, editors. GeneReviews (R). Seattle (WA); 1993.

[17] Sissler M, Gonzalez-Serrano LE, Westhof E. Recent advances in mitochondrial aminoacyl-tRNA synthetases and disease. Trends Mol Med 2017;23:693–708. 28716624 10.1016/j.molmed.2017.06.002

[18] Boczonadi V, Jennings MJ, Horvath R. The role of tRNA synthetases in neurological and neuromuscular disorders. FEBS Lett 2018;592:703–17. 29288497 10.1002/1873-3468.12962 PMC5873386

[19] Scheper GC, van der Klok T, van Andel RJ, van Berkel CG, Sissler M, Mitochondrial aspartyl-tRNA synthetase deficiency causes leukoencephalopathy with brain stem and spinal cord involvement and lactate elevation. Nat Genet 2007;39:534–9. 17384640 10.1038/ng2013

[20] Konovalova S, Tyynismaa H. Mitochondrial aminoacyl-tRNA synthetases in human disease. Mol Genet Metab 2013;108:206–11. 23433712 10.1016/j.ymgme.2013.01.010

[21] Webb BD, Wheeler PG, Hagen JJ, Cohen N, Linderman MD, Novel, compound heterozygous, single-nucleotide variants in MARS2 associated with developmental delay, poor growth, and sensorineural hearing loss. Hum Mutat 2015;36:587–92. 25754315 10.1002/humu.22781 PMC4439286

[22] Pierce SB, Chisholm KM, Lynch ED, Lee MK, Walsh T, Mutations in mitochondrial histidyl tRNA synthetase HARS2 cause ovarian dysgenesis and sensorineural hearing loss of Perrault syndrome. Proc Natl Acad Sci U S A 2011;108:6543–8. 21464306 10.1073/pnas.1103471108 PMC3081023

[23] Pierce SB, Gersak K, Michaelson-Cohen R, Walsh T, Lee MK, Mutations in LARS2, encoding mitochondrial leucyl-tRNA synthetase, lead to premature ovarian failure and hearing loss in Perrault syndrome. Am J Hum Genet 2013;92:614–20. 23541342 10.1016/j.ajhg.2013.03.007 PMC3617377

[24] Belostotsky R, Ben-Shalom E, Rinat C, Becker-Cohen R, Feinstein S, Mutations in the mitochondrial seryl-tRNA synthetase cause hyperuricemia, pulmonary hypertension, renal failure in infancy and alkalosis, HUPRA syndrome. Am J Hum Genet 2011;88:193–200. 21255763 10.1016/j.ajhg.2010.12.010 PMC3035710

[25] Antonellis A, Ellsworth RE, Sambuughin N, Puls I, Abel A, Glycyl tRNA synthetase mutations in Charcot-Marie-Tooth disease type 2D and distal spinal muscular atrophy type V. Am J Hum Genet 2003;72:1293–9. 12690580 10.1086/375039 PMC1180282

[26] McLaughlin HM, Sakaguchi R, Liu C, Igarashi T, Pehlivan D, Compound heterozygosity for loss-of-function lysyl-tRNA synthetase mutations in a patient with peripheral neuropathy. Am J Hum Genet 2010;87:560–6. 20920668 10.1016/j.ajhg.2010.09.008 PMC2948804

[27] Santos-Cortez RL, Lee K, Azeem Z, Antonellis PJ, Pollock LM, Mutations in KARS, encoding lysyl-tRNA synthetase, cause autosomal-recessive nonsyndromic hearing impairment DFNB89. Am J Hum Genet 2013;93:132–40. 23768514 10.1016/j.ajhg.2013.05.018 PMC3710764

[28] Ruzzenente B, Assouline Z, Barcia G, Rio M, Boddaert N, Inhibition of mitochondrial translation in fibroblasts from a patient expressing the KARS p.(Pro228Leu) variant and presenting with sensorineural deafness, developmental delay, and lactic acidosis. Hum Mutat 2018;39:2047–59. 30252186 10.1002/humu.23657

[29] Ardissone A, Tonduti D, Legati A, Lamantea E, Barone R, KARS-related diseases: progressive leukoencephalopathy with brainstem and spinal cord calcifications as new phenotype and a review of literature. Orphanet J Rare Dis 2018;13:45. 29615062 10.1186/s13023-018-0788-4 PMC5883414

[30] Scheidecker S, Bar S, Stoetzel C, Geoffroy V, Lannes B, Mutations in KARS cause a severe neurological and neurosensory disease with optic neuropathy. Hum Mutat 2019;40:1826–40. 31116475 10.1002/humu.23799

[31] Verrigni D, Diodato D, Di Nottia M, Torraco A, Bellacchio E, Novel mutations in KARS cause hypertrophic cardiomyopathy and combined mitochondrial respiratory chain defect. Clin Genet 2017;91:918–23. 27891585 10.1111/cge.12931

[32] Kohda M, Tokuzawa Y, Kishita Y, Nyuzuki H, Moriyama Y, A comprehensive genomic analysis reveals the genetic landscape of mitochondrial respiratory chain complex deficiencies. PLoS Genet 2016;12:e1005679. 26741492 10.1371/journal.pgen.1005679 PMC4704781

[33] Friederich MW, Timal S, Powell CA, Dallabona C, Kurolap A, Pathogenic variants in glutamyl-tRNA(Gln) amidotransferase subunits cause a lethal mitochondrial cardiomyopathy disorder. Nat Commun 2018;9:4065. 30283131 10.1038/s41467-018-06250-w PMC6170436

[34] Fine AS, Nemeth CL, Kaufman ML, Fatemi A. Mitochondrial aminoacyl-tRNA synthetase disorders: an emerging group of developmental disorders of myelination. J Neurodev Disord 2019;11:29. 31839000 10.1186/s11689-019-9292-y PMC6913031

[35] D’Souza AR, Minczuk M. Mitochondrial transcription and translation: overview. Essays Biochem 2018;62:309–20. 30030363 10.1042/EBC20170102 PMC6056719

[36] Amunts A, Brown A, Toots J, Scheres SHW, Ramakrishnan V. Ribosome. The structure of the human mitochondrial ribosome. Science 2015;348:95–8. 25838379 10.1126/science.aaa1193 PMC4501431

[37] Greber BJ, Bieri P, Leibundgut M, Leitner A, Aebersold R, Ribosome. The complete structure of the 55S mammalian mitochondrial ribosome. Science 2015;348:303–8. 25837512 10.1126/science.aaa3872

[38] Hakli S, Luotonen M, Sorri M, Majamaa K. Mutations in the two ribosomal RNA genes in mitochondrial DNA among Finnish children with hearing impairment. BMC Med Genet 2015;16:3. 25650108 10.1186/s12881-015-0145-6 PMC4410458

[39] Zhao H, Li R, Wang Q, Yan Q, Deng JH, Maternally inherited aminoglycoside-induced and nonsyndromic deafness is associated with the novel C1494T mutation in the mitochondrial 12S rRNA gene in a large Chinese family. Am J Hum Genet 2004;74:139–52. 14681830 10.1086/381133 PMC1181901

[40] Kullar PJ, Gomez-Duran A, Gammage PA, Garone C, Minczuk M, Heterozygous SSBP1 start loss mutation co-segregates with hearing loss and the m.1555A>G mtDNA variant in a large multigenerational family. Brain 2018;141:55–62. 29182774 10.1093/brain/awx295 PMC5837410

[41] Alila-Fersi O, Chamkha I, Majdoub I, Gargouri L, Mkaouar-Rebai E, Co segregation of the m.1555A>G mutation in the MT-RNR1 gene and mutations in MT-ATP6 gene in a family with dilated mitochondrial cardiomyopathy and hearing loss: a whole mitochondrial genome screening. Biochem Biophys Res Commun 2017;484:71–8. 28104394 10.1016/j.bbrc.2017.01.070

[42] Bianco A, Bisceglia L, De Caro MF, Galeandro V, De Bonis P, Leber’s hereditary optic neuropathy, intellectual disability and epilepsy presenting with variable penetrance associated to the m.3460G >A mutation and a heteroplasmic expansion of the microsatellite in MTRNR1 gene - case report. BMC Med Genet 2018;19:129. 30053855 10.1186/s12881-018-0644-3 PMC6062935

[43] Liu Z, Song Y, Li D, He X, Li S, The novel mitochondrial 16S rRNA 2336T>C mutation is associated with hypertrophic cardiomyopathy. J Med Genet 2014;51:176–84. 24367055 10.1136/jmedgenet-2013-101818 PMC3932983

[44] Lake NJ, Webb BD, Stroud DA, Richman TR, Ruzzenente B, Biallelic mutations in MRPS34 lead to instability of the small mitoribosomal subunit and leigh syndrome. Am J Hum Genet 2017;101:239–54. 28777931 10.1016/j.ajhg.2017.07.005 PMC5544391

[45] Chen A, Tiosano D, Guran T, Baris HN, Bayram Y, Mutations in the mitochondrial ribosomal protein MRPS22 lead to primary ovarian insufficiency. Hum Mol Genet 2018;27:1913–26. 29566152 10.1093/hmg/ddy098 PMC5961111

